# ‘I’m unlikeable, boring, weird, foolish, inferior, inadequate’: how to address the persistent negative self-evaluations that are central to social anxiety disorder with cognitive therapy

**DOI:** 10.1017/S1754470X22000496

**Published:** 2022-12-01

**Authors:** Emma Warnock-Parkes, Jennifer Wild, Graham Thew, Alice Kerr, Nick Grey, David M. Clark

**Affiliations:** 1 Department of Experimental Psychology, University of Oxford, Oxford, UK; 2 Oxford Health NHS Foundation Trust, Oxford, UK; 3 Department of Psychology, King’s College London, London, UK; 4 Sussex Partnership NHS Foundation Trust, Worthing, UK

**Keywords:** CBT, cognitive therapy, low self-esteem, negative self-beliefs, social anxiety disorder, unconditional beliefs

## Abstract

Patients with social anxiety disorder (SAD) have a range of negative thoughts and beliefs about how they think they come across to others. These include specific fears about doing or saying something that will be judged negatively (e.g. ‘I’ll babble’, ‘I’ll have nothing to say’, ‘I’ll blush’, ‘I’ll sweat’, ‘I’ll shake’, etc.) and more persistent negative self-evaluative beliefs such as ‘I am unlikeable’, ‘I am foolish’, ‘I am inadequate’, ‘I am inferior’, ‘I am weird/different’ and ‘I am boring’. Some therapists may take the presence of such persistent negative self-evaluations as being a separate problem of ‘low self-esteem’, rather than seeing them as a core feature of SAD. This may lead to a delay in addressing the persistent negative self-evaluations until the last stages of treatment, as might be typically done in cognitive therapy for depression. It might also prompt therapist drift from the core interventions of NICE recommended cognitive therapy for social anxiety disorder (CT-SAD). Therapists may be tempted to devote considerable time to interventions for ‘low self-esteem’. Our experience from almost 30 years of treating SAD within the framework of the Clark and Wells (1995) model is that when these digressions are at the cost of core CT-SAD techniques, they have limited value. This article clarifies the role of persistent negative self-evaluations in SAD and shows how these beliefs can be more helpfully addressed from the start, and throughout the course of CT-SAD, using a range of experiential techniques.

**Key learning aims:**

1. To recognise persistent negative self-evaluations as a key feature of SAD.
2. To understand that persistent negative self-evaluations are central in the Clark and Wells (1995) cognitive model and how to formulate these as part of SAD.
3. To be able to use all the experiential interventions in cognitive therapy for SAD to address these beliefs.

## Introduction

Cognitive therapy for social anxiety disorder (CT-SAD) based on the Clark and Wells’ (1995) model is recommended by NICE as a first line intervention. The treatment focuses on helping patients overcome social anxiety-related negative thinking and associated patterns of attention and behaviour. This article clarifies how the treatment is used with a set of negative thoughts and beliefs whose role in social anxiety disorder (SAD) is sometimes misunderstood.

People with SAD fear that when they are in a social situation they will do or say something that will be embarrassing or humiliating (*DSM-5*; American Psychiatric Association, 2013). Clinically we observe that the thoughts patients have in social situations are a mixture of predictions about things they fear *will happen* and judgements of things they think *have already happened*. For example, ‘I will blush’ and ‘I am blushing’; ‘People will think I am being boring’ and ‘I’m boring’. All these thoughts revolve around negative evaluation of one’s social self. They reflect a strong desire to convey a favourable impression of oneself to others, and marked insecurity about one’s ability to do so. Some of the thoughts represent a fear of showing signs of anxiety that people with SAD may think are unacceptable (e.g. tremble or shake uncontrollably; be paralysed with fear; drop or spill things; be sick; babble or talk funny; be unable to concentrate; be unable to write properly; sweat or perspire; blush; look nervous). Other negative self-evaluative thoughts do not explicitly refer to signs of anxiety. Typical thoughts in this category are: ‘I am unlikeable’, ‘I am foolish’, ‘I am inadequate’, ‘I am inferior’, ‘I am weird/different’ and ‘I am boring’. Such thoughts have a global negative feel to them. We have noticed that some therapists take the presence of such thoughts as an indication that treatment should focus on ‘low self-esteem’, even when the thoughts largely revolve around social interactions. This can mean that treatment of an individual with marked social anxiety digresses into devoting considerable time in early sessions to using cognitive techniques that are common in low self-esteem work (e.g. continua, positive data logs, automatic thought records). Others may follow the typical order of cognitive therapy for depression and not explicitly address the global sounding negative evaluations until the later stages of therapy. When treating a person who is requesting help with SAD, our experience is that neither of these therapist manoeuvres is helpful. Thoughts such as ‘I am unlikeable’, ‘I am inferior’ and ‘I am boring’ are just as much a core part of SAD as thoughts such as ‘I am blushing’. They are all part of the evaluation of how people with SAD think they appear to others (their social self). These persistent negative self-evaluations should therefore be brought into play from the start of CT-SAD and be addressed with each of the core techniques of the social anxiety treatment.

For some people, negative evaluation of one’s social self has a particularly harsh tone, with thoughts such as ‘I’m boring’, ‘I’m inadequate’, ‘I’m weird’, being experienced as a self-attack. Patients’ self-criticism can be an echo from the past; for example, the internalised voice of a critical or abusive caregiver. However, for others there is no clear origin. Whatever the origin of the harsh, putting-down tone, some therapists take the presence of such self-criticism as an indication that the core techniques of cognitive therapy for SAD are not appropriate and digress to other types of intervention. Once again, our experience is that such digression can be unhelpful if it unnecessarily delays or prevents delivery of key CT-SAD interventions. We describe some additional manoeuvres that can help take the sting out of the self-criticism. We would argue that it is usually best to work through the usual stages of the CT-SAD protocol, augmented by these techniques when needed.

In this article, we outline how to ensure that persistent negative self-evaluations are addressed in CT-SAD, by ensuring they are ‘put on the table’ throughout therapy, at the same time as one addresses negative thoughts about showing signs of anxiety. This is a position about the optimal conduct of therapy which has evolved gradually in our group in the light of almost 30 years of experience in treating SAD within the framework of the Clark and Wells (1995) cognitive model. We hope therapists will find our detailed description of how to address these beliefs at each stage of CT-SAD helpful. To aid assessment and treatment planning, we also include a brief section on working with co-morbid depression at the end of this paper. However, before doing that, we thought it would be helpful to briefly summarise the Clark and Wells (1995) cognitive model of social anxiety, placing particular emphasis on the relationship between assumptions and negative self-evaluative thoughts, and on the role of attention and safety behaviours in maintaining patients’ fears.

### Clark and Wells’ cognitive model of SAD

People with SAD have a strong desire to convey a favourable impression of themselves to others, but feel very insecure about their ability to do so. Many of the things that happen in a social situation are ambiguous and could be interpreted in several ways. For example, if two people are having a conversation and one person looks out of the window, there could be a range of neutral, positive or negative interpretations (e.g. ‘they have seen something interesting outside’, ‘I’m so clever they are having to think carefully about what I say’, ‘they think I am boring them’). Patients with SAD tend to jump to a negative interpretation of such an ambiguous social event. They also tend to interpret mildly negative social events/comments in a catastrophic fashion (Stopa and Clark, 1993). Within the cognitive model, such interpretations are seen as arising from a series of negative beliefs or assumptions that people with SAD hold about their social self and the nature of social interactions. Clark and Wells (1995) suggested that these assumptions can be divided into three groups. The first two groups are unhelpful rules that people feel they should follow to be accepted by others:

- **Excessively high standards for social performance.** For example, ‘I should be intelligent and witty’, ‘My speech should be perfectly fluent’, ‘I must never show signs of anxiety’. These rules assume that even casual social interactions are a performance. One must perform well to be accepted by others, rather than simply being oneself and letting people get to know you. As the standards are unrealistic, people with SAD frequently conclude they have fallen short, which will trigger thoughts such as ‘I am inadequate, boring’, etc.
- **Conditional beliefs concerning social evaluation.** These beliefs take the form, if *X* happens, then *Y* follows. For example, ‘If others want to know me, they will let me know’; ‘If I disagree with someone, they will think I am stupid’. Both beliefs would lead people to hold back in conversations, which will mean others cannot get to know them and will interact with them less. This is likely to trigger thoughts during a social interaction such as ‘I am unlikeable’.

The final set of assumptions were termed ‘unconditional beliefs about the self’ (Clark and Wells, 1995; p. 76). They were described as unconditional because they are persistent with patients endorsing them outside of social situations as well as during social situations. They are essentially patients’ conclusions about themselves based on prior experience in social interactions. Once established, these **persistent negative self-evaluations** (as we refer to them in this paper) make it much more likely that the individual will jump to a negative conclusion about their performance in a future social situation. For example, somebody who holds the general belief that ‘I am a boring person’ will be more likely to interpret an absence of positive feedback during a conversation as evidence that they are being boring at that moment, triggering the negative automatic thought: ‘I’m boring’.

Once patients start having negative thoughts about their performance in social situations, several key processes come into play that maintain their negative social thoughts and assumptions. The first of these processes is **self-focused attention**. In social situations, patients’ focus of attention shifts from being on the external social interaction (e.g. listening to what people are saying, being lost in the conversation) to being more internally focused (e.g. focusing on their internal feelings of anxiety, monitoring how they are coming across). This self-focused attention, like a spotlight turning on the self, heightens awareness of anxious thoughts and feelings and can feed into a **negative self-image or impression** of how the person is coming across. For example, somebody who is overly focused on how hot they feel internally pictures themselves looking sweaty and coming across as weird to others. Somebody who spends all their interactions monitoring how boring they think they sound has an impression of themselves looking dull and distant from others. When patients are paying so much attention to themselves, their social performance, and negative self-images/impressions, they often fail to notice any positive reactions from their conversational partners. They are more likely to use their internal information to infer judgements of the social interaction, which contributes to more negative self-evaluations. For example, if a patient thinks of himself as unlikeable and inferior and is focused on how he is coming across and his feelings of inferiority, he will fail to notice the person speaking to him seems interested in what he has to say. Instead, he will use his anxious feelings as an indication that he was coming across badly and therefore assume that other people are looking down on him.

The second process that maintains negative thoughts and assumptions is the **safety behaviours** that patients use to avoid revealing aspects of themselves that they believe are unacceptable and to prevent their feared predictions from happening. For example, a patient who fears that they are unlikeable might keep quiet during conversations, stay on the edge of groups, and share little of themselves. These strategies will limit opportunities for positive interactions, make it hard for patients to form close and meaningful relationships with others, and thus maintain their view of themselves as being unlikeable. In addition to avoidant and self-concealment strategies, therapists need to look out for impression management strategies, which can be harder to spot. For example, the same patient rehearses likeable and entertaining things to say before meeting colleagues. While she is running through these topics in the meeting, she may appear confident and outgoing. However, her belief that she is unlikeable is unlikely to change even if the conversation is going well, because she will think she only prevented people from discovering that she is unlikeable because she rehearsed topics to say in advance. She is likely to feel that without such rehearsal, people would have realised that she is unlikeable. Both avoidant and impression management strategies are ways of hiding the self from the world and thus maintain negative self-beliefs, preventing patients from discovering that they are acceptable to other people.

## How persistent negative self-evaluations are addressed in CT-SAD

### All components of CT-SAD are designed to experientially target persistent negative self-evaluations

CT-SAD (Clark and Wells, 1995; Clark *et al*., 2003; Clark *et al*., 2006) is derived from the cognitive model and is typically delivered in up to fourteen 90-minute weekly sessions over 3–4 months. Treatment can be delivered in person or remotely via video conferencing (see Warnock-Parkes *et al*., 2020) and an effective internet-delivered version of the treatment has also been developed (Clark *et al*., 2022). A key goal of treatment is to help patients discover that when they are themselves and do not hide away or put on a front, they are acceptable to others. To achieve this, therapy needs to identify and target all the processes that maintain patients’ anxiety, including negative thoughts and assumptions/beliefs, self-focused attention, distorted self-images/impressions, safety behaviours and self-criticism. Discussion techniques alone are unlikely to achieve reduction in these core maintaining processes, so will be limited in their effectiveness for belief change. Therefore, the architecture of CT-SAD is a range of experiential exercises within the framework of belief change. The experiential nature of the treatment provides concrete evidence for change in negative beliefs. This is particularly important when addressing persistent negative self-evaluations (e.g. likeability, acceptability, weirdness) which are often abstract and subjective concepts, so difficult to change with discussion alone. Videos demonstrating how to deliver each of the interventions that are outlined are available on our free therapist resources website (www.oxcadatresources.com), alongside treatment manuals and key clinical papers.

### Eliciting persistent negative self-evaluations in the CT-SAD formulation

**Figure 1.**
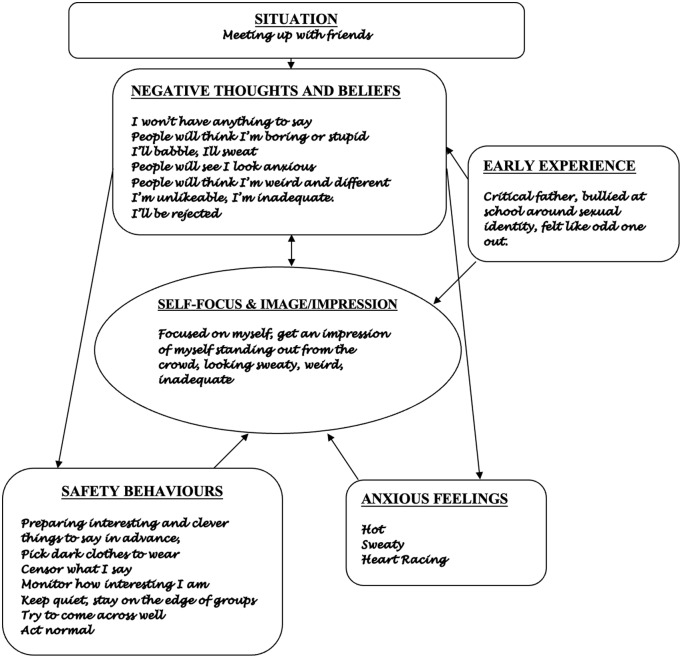
Example formulation for somebody with SAD.

After an initial clinical assessment, the first step in CT-SAD is to identify some goals and develop an individualised version of the cognitive model that acts as a roadmap for therapy. An example is given in Fig. 1. Therapist and patient work together to map out what happened when the person was anxious in a recent social situation. As illustrated in Fig. 1, patients usually volunteer negative thoughts about what they feared they might do or say (e.g. ‘I won’t have anything to say’, ‘I’ll babble’, ‘I’ll sweat’). Exploring ‘*what would be so bad about that?*’ can lead to clues about patient’s negative self-evaluations (e.g. as shown in Fig. 1: ‘I’m unlikeable’, ‘I’m inadequate’, ‘I’m weird and different’). However, if patients do not report negative self-evaluations, it is worth continuing to probe for them, as in our experience most patients with social anxiety hold them. Feeling embarrassed or ashamed about reporting upsetting beliefs such as ‘I’m weird’ and ‘I’m unlikeable’ can be a roadblock to expressing them. Furthermore, as persistent negative self-evaluations have often been present for many years, these can act like a silent assumption or a felt sense, ever present yet hard to easily identify. Therapists could ask a normalising question, for example, ‘*Many people who feel anxious in social situations find they sometimes have upsetting thoughts or feelings about themselves socially, for example seeing themselves as unlikeable, weird, or inadequate, etc. Did you have any of these kinds of thoughts about yourself in this situation? Or have you at other times?*’.

### Questionnaires

#### Questionnaires are a powerful tool in targeting persistent negative self-evaluations in treatment

Process measures such as the Social Cognitions Questionnaire (SCQ; Clark, 2005) and the Social Attitudes Questionnaire (SAQ; Clark, 2005) are also helpful in spotting persistent negative self-evaluations. They can be used to amplify the information gathered when mapping the formulation and to review change in these beliefs across therapy. The SCQ, used to assess several thoughts and beliefs, includes items such as ‘*I’m unlikeable*’, ‘*I am foolish*’, ‘*I am inadequate*’, ‘*I am inferior*’, ‘*I am vulnerable*’, ‘*I’m weird/different*’. The SAQ includes a range of assumptions including: ‘*I’m unlikeable*’, ‘*I’m different*’, ‘*I’m unacceptable*’, ‘*I’m inferior*’, ‘*I’m vulnerable*’, ‘*I’m a weird person*’, ‘*I’m odd/peculiar*’, ‘*I’m inadequate*’ and ‘*I’m a boring person*’. These beliefs are then added to the formulation (see Fig. 1), even if they are identified predominantly from discussion of the questionnaires.

We also give the Social Behaviour Questionnaire (SBQ; Clark, 2005) at the start, middle and end of treatment, as ongoing use of safety behaviours is one of the key processes that prevents people from disconfirming their negative predictions and changing their negative self-beliefs. This measure can therefore also be used to helpfully inform the formulation.

The SCQ, SBQ and SAQ are available at www.oxcadatresources.com.

### Target persistent negative self-evaluations early! – Using the self-focused attention and safety behaviours experiment with video feedback

The first two experiential exercises in CT-SAD (self-focused attention and safety behaviours experiment and video feedback) can both be used to help patients question their persistent negative self-evaluative beliefs. In the self-focused attention and safety behaviours experiment patients are often encouraged to have a conversation with a stranger twice, once while focusing their attention on themselves and doing some of their safety behaviours and then while attempting to shift to an external focus of attention and drop their safety behaviours. The aim of this experiment is to help patients discover that the things they are understandably doing to manage social anxiety are inadvertently fuelling the problem. In particular, focusing attention on themselves and doing their safety behaviours makes them feel more anxious, gives them the impression that they come across less well to the other person, and prevents them from disconfirming their negative beliefs. Furthermore, the two interactions are recorded, and subsequent viewing of the video is used to help patients discover that they come across more positively than they think. These two exercises together establish that the notion of coming across as unlikeable/weird/inferior is an idiosyncratic self-evaluation, it is not how the world sees the patient, but is instead a view of themselves maintained by self-focused attention and safety behaviours. Full descriptions and video role-plays of these techniques are available on the oxcadatresources.com website. Tips for weaving in and targeting persistent negative self-evaluations in these early experiential exercises are outlined below.

### The self-focused attention and safety behaviours experiment

#### Identifying relevant negative self-evaluations and incorporating them as predictions

To target negative self-evaluations from the start of therapy they need to be ‘put on the table’. At the start of the experiment, patients are asked what key fears they have about a brief social interaction with a stranger and what associated safety behaviours they would use. As mentioned above, patients do not always spontaneously volunteer negative evaluations, so therapists should ask about them if relevant, incorporating them as predictions in the experiment. For example, ‘*I can see on the Social Cognitions Questionnaire/the model we drew out last week that you worry about coming across as unlikeable, is this a concern you would have speaking to a stranger today?*’.

#### ‘The real threat is in my head’: maximising learning about negative self-beliefs

After patients have completed each of the two social interactions various (0–100%) ratings are taken (How much did the person use their safety behaviours? How self-focused were they? How anxious did they feel? How much did they believe all their key fears and concerns?). These are compared in a two-column table. When reflecting on the conversations and looking with the therapist at the ratings they made about both interactions, patients usually discover that focusing externally and dropping safety behaviours leads them to feel less anxious and they think they come across better. Further discussion can help patients to see that their negative self-evaluations are their own perceptions projected into the minds of others; that they are more acceptable to others than they think they are. For example, ‘*So when you are more in your own head and monitoring yourself more, you think you come across worse to other people – what do you make of that?*’; ‘*How did the other person actually respond to you? Did they respond differently? Did they respond as if you were unlikeable or weird?*’.

### Seeing is believing: using video and still-image feedback

Video feedback of the interactions recorded during the self-focused attention and safety behaviours experiment is usually done in the next treatment session. Patients are encouraged to make several predictions in advance of viewing video based on their feelings about how they might come across. Comparing the patient’s ratings made before and after viewing video usually facilitates the discovery that patients come across much better than they predicted based on their feelings, and sometimes even better when they were more externally focused and not using their safety behaviours. Patients who have negative self-evaluations are likely to benefit significantly from video feedback if their negative beliefs are identified as predictions ahead of viewing video. However, these patients may also be prone to several processing biases that might limit the benefits from this technique if it is not set up in a careful and considered manner. For example, patients can be prone to habitual self-criticism, so might selectively search for behaviours that could be interpreted negatively or re-experience feelings when watching the video. For a clinical guide on delivering video feedback and overcoming all the common processing biases, see Warnock-Parkes *et al*. (2017) and the associated materials that are available at www.oxcadatresources.com.

#### What does ‘unlikeable’ look like? Operationalising persistent negative self-evaluations

Patients’ persistent negative beliefs about the way they appear to others (e.g. ‘*I’m weird, I’m inferior, I’m unlikeable*’) have often not been operationalised. They may feel inferior or weird but struggle to say what that would look like when asked. Spending a little time trying to be concrete about how they would appear to someone else if they were weird or inferior can therefore be very helpful. Once this is clarified, patients can view the video to see whether they really come across in the way they fear. For example:

**Therapist:** If you were to come across as 90% unlikeable as you predicted, what would we see on video?

**Patient:** I’m not sure, I just feel that way.

**Therapist:** That is interesting, that you feel sure you come across as unlikeable, but not sure what an unlikeable person looks like. When talking to other people do you often decide they are unlikeable?

**Patient:** No, not at all.

**Therapist:** Is it possible that this is mainly the way you think about yourself rather than something that is in the mind of others?

**Patient:** I guess it could be.

**Therapist:** Well, I wonder if we could find out more about that by watching the video. How might we see the other person respond if they thought you were unlikeable?

Obtaining observable predictions, e.g. ‘*If I were to come across as 80% weird, I will see the other person giving a weird look, rolling their eyes, trying to end the chat early*’, maximises the chance of belief disconfirmation, as patients usually observe that the other person they spoke to responded as if they are a normal, acceptable person.

#### Seeing on video that ‘I’m an acceptable person, like anybody else’

The aim in video feedback is to help patients see themselves through the eyes of an objective other person, rather than through the lens of their negative self-evaluations. To help switch off patients’ habitual self-criticism and get them into a more objective mode for viewing the video, the therapist encourages the patient to ‘*watch the screen like you would a television show, as if you are watching two strangers. Look at everybody on screen, not just one person*’. It helps if therapists purposefully refer to the patient as ‘that person’ when discussing the footage, rather than using their name. It can also help to play the recording with the sound turned off for a few seconds, as this tends to help patients get into a less critical mode of viewing. When video feedback is set up in this way, it is a powerful technique to help patients discover early in therapy that, contradictory to their negative self-evaluations, that they are acceptable, just like anybody else. This can often be achieved by turning the sound off and watching the conversation for a few seconds while asking the patient:

**Therapist:** If you walked into a coffee shop and saw those two people talking, what would your general impression of the conversation be?

**Patient:** I guess it looks ok, kind of normal.

**Therapist:** Looking at these two people, do you think that person (points to the conversational partner) seems acceptable and likeable?

**Patient:** Yes of course.

**Therapist:** So, looking at the person she is talking to (points to the patient), does that person look notably different?

**Patient:** No, I guess not.

**Patient:** Would either of those two people stand out to you as looking 80% weird or 70% unlikeable?

**Patient:** Er, no I guess not, it just looks like a normal chat.

**Therapist:** That is interesting because we know that one of those people feels like they look 80% weird and 70% unlikeable – but if we didn’t know either of those people would we have any idea who it was that was feeling like that?

The patient could also be asked ‘*If an alien came from space would they think something looked odd in this conversation? Would they say one of these humans was acceptable and the other wasn’t? Why not?*’.

Patients may have moments when viewing that cause them concern because they think they have revealed something about themselves they fear others would find unacceptable, (e.g. a patient thinks they blushed noticeably without hiding their face and this came across as weird; or they spoke spontaneously without preparing interesting topics and think they said something boring or unlikeable). If this happens during video feedback it is an excellent opportunity for belief disconfirmation. It can help to pause and rewind the video to the moment of concern, helping the patient to switch their attention when watching these moments back to how the other person is responding:

**Therapist:** So, let’s rewind to the moment you think you said something stupid and were coming across as inferior. Let’s pause here and look at the other person on the video. How are they reacting to that man (points to the patient)?

**Patient:** Err… they are just smiling.

**Therapist:** Are they giving him a weird, condescending look, as if he were stupid and inferior, as you predicted?

**Patient:** I guess not. They are just acting normally.

**Therapist:** Normally. Interesting. So even though that man is feeling as though he is coming across as 80% stupid and inferior, the other person is responding normally.

What does that tell us about this man and how acceptable he is?

**Patient:** I guess maybe he is more acceptable than he thinks, he isn’t as stupid as he feels.

### Capturing moments of belief disconfirmation: still-image flashcards

**Figure 2.**
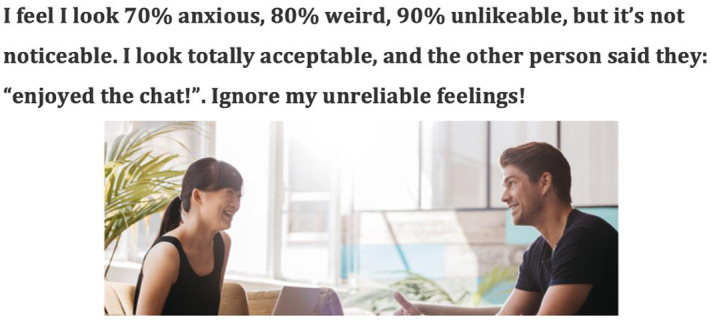
A still-image flashcard used to update persistent negative self-evaluations.

Capturing still images from these key moments of discovery during video feedback is a helpful way to develop a more accurate image of the patient’s social self that contradicts their own negative self-evaluation. For example, the moment when the conversational partner is clearly enjoying the chat and both people are smiling is captured for a person who fears being unlikeable, or the moment when a patient felt they said something particularly weird and the person they are speaking to is smiling and does not appear to have noticed anything. Key learning points can be added to these images to create a helpful flashcard that can be printed out or stored on the patient’s phone and reviewed over the week (see Fig. 2). More examples are given in our clinical guide to video feedback (see Warnock-Parkes *et al*., 2017).

### Using other person feedback to get further change in persistent negative self-evaluations

Further shift in beliefs and confirmation of the discoveries made while viewing the video is often possible by obtaining written feedback from the other person in the interactions. This feedback tends to confirm and strengthen the more positive view of their social self that patients have gained from watching the video. We include a template that could be used to obtain other person feedback on the www.oxcadatresources.com website. From looking at what other people made of the conversations (e.g. ‘I enjoyed the chat, Samira was friendly’) and examining ratings given of the patients’ key fears and concerns compared with their own ratings, patients usually see that although they rate themselves as coming across as 80% weird or 90% unlikeable, other people give significantly lower ratings, usually 0%.

The stark difference is discussed to lead to further change in beliefs. ‘*You see yourself as 80% weird and unlikeable but they don’t mention those things at all in their initial impressions of the conversations – what do you make of that?*’; ‘*How does the feedback fit with your view of yourself as weird and unlikeable?*’; ‘*What does their ratings tell you about how acceptable you are?*’.

### Shifting self-focused attention is key to discovering you are acceptable

Patients are encouraged to be externally focused during behavioural experiments carried out in CT-SAD, as this makes it easier for them to discover that they are acceptable when they interact with other people without doing their safety behaviours. To help patients become externally focused we typically include a session of attention training exercises after the video feedback session and encourage patients continue practising external focus for homework. A video illustrating attention training is available on the oxcadatresources.com website. As patients become more externally focused, their negative self-perceptions tend to become less prominent.

### Experiment! Experiment! Experiment!

#### Dropping safety behaviours: only by revealing our true selves can we discover we are acceptable to others

Behavioural experiments are a key belief change technique and are used in most CT-SAD sessions and homework. The overall framework for behavioural experiments is cognitive change, rather than habituation. Patients engage in feared interactions/situations having made predictions in advance about what they think is the worst that could happen if they were to drop their self-focus and safety behaviours. For example, a patient predicts ‘If I take part in the conversation without trying to come across well or preparing interesting things to say, people will think I am unlikeable and ignore me’. The key aim is for patients to discover for themselves that they are acceptable when they drop their safety behaviours and are themselves. In addition, they are likely to discover that they can trust themselves to come across well without having to do any special safety manoeuvres or carefully monitoring themselves. This experiential discovery provides a more powerful way to disconfirm persistent negative self-evaluations than by discussion alone. Video, still-image and other person feedback can also be used throughout several in-session behavioural experiments to maximise belief change. When setting up behavioural experiments it is important to not just focus on specific fearful thoughts about things one might do or say (e.g. ‘I won’t have anything to say’, ‘I’ll blush’, etc.). If persistent negative self-evaluative beliefs are also endorsed on the SCQ they should be brought into the predictions and plans for setting up the behavioural experiment, when relevant.

*Negative self-evaluations are addressed at every stage of planning and carrying out experiments:*

1. ***Initial discussion to loosen beliefs***, e.g. ‘What have we already learnt in treatment about the belief you are unlikeable [insert patient’s key problematic beliefs]?’
2. ***Developing observable predictions and rating these***, e.g. ‘How much do you believe the other people will think you are unlikeable/weird/foolish in this conversation if you drop your safety behaviours and are just being yourself?’; ‘How would we know this? What reactions would we observe?’
3. ***Targeting key safety behaviours to change within the chosen situation***: ‘What are the key safety behaviours you use when you are thinking somebody might think you are weird/odd/unlikeable?’; ‘What will you do differently in this experiment?’
4. ***Subsequently reviewing the outcome based on what happened, not feelings***, e.g. ‘Putting how you felt aside, what did you observe during that conversation?’; ‘How did the others respond?’; ‘Did they respond as if you were 80% unlikeable?’
5. ***Generating generalised learning***, e.g. ‘If they didn’t respond to you as if you are unlikeable, how did they actually respond to you?’; ‘What does this tell you about how you come across in social situations more generally?’; ‘Is it possible you come across as more acceptable than you think in general?; ‘What might this tell you about yourself more widely?’; ‘How can you remind yourself of this?’
6. ***Building on learning by testing beliefs further***: ‘How can we test this out further?’; ‘What experiments could you try over the week to build on our learning about this?’

##### Addressing common blocks to generating generalised learning from experiments

When discussing learning from experiments, patients with SAD can be reluctant to state more positive alternative beliefs like ‘I’m an acceptable person’ aloud. In these cases, it is worth asking what thoughts might be getting in the way. A common blocking belief is that the patient will become or appear ‘boastful’ or ‘big-headed’. This can usually be easily addressed by exploring ‘*Given you see yourself as 80% unlikeable and have done for most of your life, how likely is it that by doing X/Y in treatment you will suddenly turn from thinking of yourself as 80% unlikeable to thinking you are the best person in the world and start boasting to others?*’. Patients are usually able to see that the work they are doing in therapy is more likely to get them along the continuum of thinking of themselves negatively to acceptably, and highly unlikely to tip them into over-confidence.

### Accumulating the data

When patients have persistent negative self-evaluations, multiple experiences of evidence to the contrary of their self-view can be needed to lead to significant belief change. As CT-SAD progresses, patients keep records of their behavioural experiments to accumulate data demonstrating they are acceptable when they drop their safety behaviours and are just being themselves. In our experience accumulating the data in this way can be particularly helpful. Reviewing video footage of several behavioural experiments, and gathering still-images that capture key moments of belief disconfirmation taken across therapy (both in session and for homework where possible), is another powerful way to accumulate concrete evidence that the person is acceptable when they are just being themselves. Below we describe two key CT-SAD strategies that we have developed over time to accumulate data disconfirming negative self-beliefs and building a view of the self as acceptable: using golden opportunities and zero avoidance week.

#### Using golden opportunities to generate data in multiple experiments

**Figure 3.**
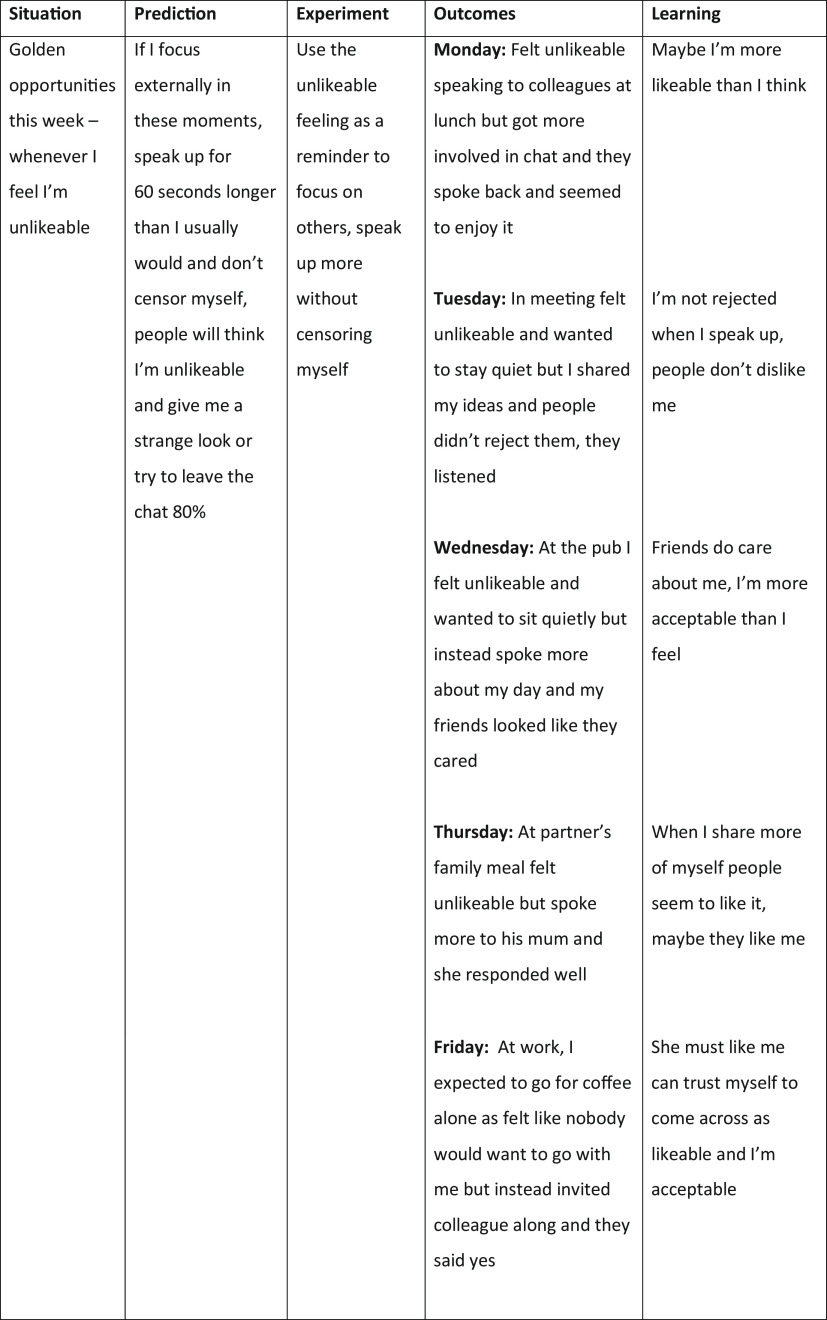
Golden opportunity experiments used to test the fear of being unlikeable.

With persistent negative self-evaluations patients usually experience a felt sense that they are vulnerable, boring, inadequate, weird, etc., multiple times a day. To maximise change in these beliefs they are encouraged to use that feeling as a cue to drop safety behaviours and switch their focus externally over the week, so they can gather and record data about how others are responding to them in these moments. These kinds of experiments can take 60 seconds or so and can happen multiple times a day, so they are a wonderful opportunity to accumulate data that patients are acceptable when they are just being themselves. Figure 3 shows a behavioural experiment record sheet used to record data from several brief golden opportunity experiments for a patient who views himself as unlikeable.

#### Zero avoidance week

Patients have often used safety behaviours to hide parts of themselves that they fear others might find unacceptable for many years. In our experience even after patients have made some significant gains in treatment, they will continue to use some of these often habitual strategies. The problem with this is that patients will only feel truly acceptable when they are able to drop these strategies and share their true selves with others. It can be useful to review questionnaires such as the SBQ at the mid-treatment point to help identify any safety behaviours patients continue to use. Once patients are aware that they are continuing to use some safety behaviours they can be encouraged to try a ‘zero avoidance week’ during therapy. This is where for a designated week (or just a day to begin with if necessary), they try to use the feeling they want to use a safety behaviour (such as keep quiet in a conversation or tell pre-prepared jokes to come across well) as a cue to do the opposite, even briefly, while observing the reactions of others. For example, if a patient tends to avoid being centre of attention for fear of being seen as weird, they are encouraged to use the feeling they want to avoid something as a cue to speak up for 60 seconds or so longer than usual and observe how others respond. Keeping a record of this, in similar way to the golden opportunities record sheet (see above) continues to accumulate data of the self as acceptable.

#### Reviewing accumulated data

Keeping behavioural experiment record sheets to accumulate the data means that patients can review learning from their experiments if more persistent negative self-evaluations are activated outside of social situations. For example, a patient who had been unemployed for many years found her beliefs (‘I’m inadequate and inferior’) were activated when searching for voluntary positions online, which led to self-critical thoughts like ‘Nobody will want you, you will make an idiot of yourself at interview’. Reading over her success experiences in her experiment logs helped her to turn her self-critical thoughts into predictions on another experiment log and fill in an application for a position that she later got.

### Taking learning a step further: decatastrophising experiments

After making progress in therapy, some patients may continue to hold onto some of their negative self-beliefs because they have the idea that they will only be accepted if they comply with certain unnecessary standards of social behaviour. For example, a patient who used to think of himself as unlikeable, but has shifted this belief during treatment, may still hold a fear if he were to strongly disagree with somebody else then that person would think of him as unlikeable and reject him. Decatastrophising experiments (sometimes referred to as ‘widening the bandwidth experiments’) are a key technique to help patients discover that even if they break the rules of how they think they must behave, they will still be accepted. It can be helpful for the therapist to model the decatastrophising experiment before asking the patient to do the same. This tends to build patients’ confidence and make it easier for them to whole-heartedly engage in decatastrophiging (e.g. saying something stupid or boring, purposefully making a mistake during a meeting at work, creating the appearance of a blush, shake or sweat).

Table 1 gives a range of example behavioural experiments, including decatastrophising experiments, that could be used to target persistent negative self-evaluations in SAD.

**Table 1.** Examples of behavioural experiments used to target different persistent negative self-evaluations

Persistent negative self-evaluations	Example experiments used to test these beliefs	Carried out in session/homework?
I’m unlikeable	Speak to a stranger. Rather than keeping quiet and holding back, contribute more in the chat, get involved	In session
	Share more of myself with colleagues over lunch without preparing things to say and monitoring how likeable I am	Homework
	Purposefully disagree with somebody during a conversation and see how they respond	In session and homework
I’m weird/different/odd	In a busy high street, rather than walking with my head down in case people are giving me weird looks, look up and observe how others respond to me	In session and homework
	Group conversation on webcam, share more about my interests and hobbies without keeping tight control of my behaviour, instead just be myself and see how they respond. Watch video back to see how they respond	In session
	Purposefully share something ‘uncool’ or not on trend in a conversation to see how they respond	In session and homework
I’m inadequate/inferior	In a small group chat rather than avoiding speaking about my work and studies as I feel I’m inferior, instead talk more about what I do and observe how others respond	In session
	Stop trying to impress/entertain other Mums in the school playground and just be myself	Homework
	Purposefully make a mistake when giving a small presentation to see how others respond	In session and homework
I’m boring	Have a webcam chat with two strangers and speak spontaneously without preparing interesting topics to talk about	In session
	Speak to friends at dinner without preparing things to talk about in advance or monitoring myself	Homework
	Purposefully talk about something I think is boring and observe reactions	In session and homework
I’m stupid/foolish	Role-play giving a report at work, instead of scripting it, speak spontaneously	In session
	Speak up in a meeting at work without trying to sound clever or censoring what I say	Homework
	Purposefully ask a stupid question, such as where the train station is, when standing near it	In session and homework

### Using surveys to loosen persistent negative self-evaluations

Surveys can be an additional technique to loosen persistent negative self-evaluations by finding out more about what other people think. For example, a therapist sent a survey to colleagues on behalf of a patient who feared they would be seen as unlikeable if they had differing opinions to others, asking: ‘*If somebody shared an opinion that differed to your own, what would you make of that? How much would you think it meant they were unlikeable 0–100%? If not, why not? What would make you decide somebody was 100% unlikeable?*’. The responses (e.g. ‘*I don’t tend to think of people as being “likeable” or “unlikeable”, but I might not get on with everybody – and some people might have traits I don’t like, for example, if they were racist, abusive or intentionally rude to me*’) helped the patient to re-think their concept of likeability and boosted their confidence to try an experiment disagreeing with others.

### Leaving the past behind: when memory-focused techniques can help address persistent negative self-evaluations

Some of the negative self-beliefs and distorted self-images/impressions patients hold are based on earlier socially traumatic experiences. For many patients with SAD, we usually find that the techniques described so far in this paper are sufficient to deal with negative self-evaluations, images and impressions. However, some patients may benefit from dealing with memories of specific socially traumatic events that appear to be linked to their persistent negative self-evaluations and distorted self-images/impressions.

### Then vs Now discrimination training

**Figure 4.**
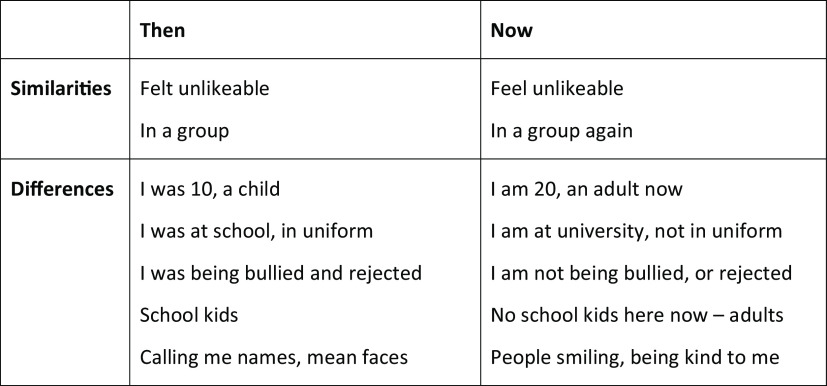
Example Then vs Now table for a patient who feels unlikeable in groups as this triggers memories of bullying.

Some patients who have experience of past social trauma tend to process present day social situations through the lens of the past. They may expect people in the present to respond to them in the same way people might have done during earlier experiences of excessive criticism, bullying, racism or victimisation. Then *vs* Now discrimination training is a technique originally developed to treat trauma triggers in post-traumatic stress disorder (Ehlers and Clark, 2000) that we have found helpful with many patients with SAD over the years. It can help patients to process current social situations without being influenced by feelings or memories from past trauma. When using Then *vs* Now, therapists help patients to spot when memories from the past might be activated and to learn to look out for key differences between THEN (their socially traumatic memories) and NOW (the present social situation). See Fig. 4 for an example of a Then *vs* Now table drawn up in therapy for a person who often felt unlikeable when around groups as this reminded her of being bullied by a group of children at primary school. A video example is given on the oxcadatresources.com website.

Incorporating this technique into practice when doing behavioural experiments in a range of social situations for homework is often successful in breaking the link between the social trauma of the past and present social situations. For example, the patient above found that actively looking out for ways people at their university were different from their bullies at school helped them to notice that they were not being put down and rejected in the present day, even though they sometimes had the felt sense of being unlikeable.

### Imagery re-scripting: changing the meaning of past social trauma

After practice using the Then *vs* Now techniques many patients find they stop re-experiencing feelings from their traumatic past during present day social situations. They are then more able to learn that others are responding to them now as if they are acceptable in their experiments. However, if memories or feelings from the past continue to intrude then we have found that imagery re-scripting, as described by Wild and Clark (2011), can be useful to change the meaning patients have attached to early socially traumatic memories, to put these in the past and achieve further improvement in persistent negative self-evaluations.

This technique is typically delivered in several steps starting with identifying a key memory that links to the patient’s present-day self-image/impression/felt sense. The therapist and patient then identify the relevant negative self-evaluations that patients have attached to the memory (e.g. I’m weird, boring, unlikeable, etc.). The therapist and patient then explore alternative evidence for the personal meaning patients have attached to their experiences. This firstly involves reviewing evidence gathered in therapy to date that does not fit with the negative meaning, e.g. ‘*What have we learnt so far in treatment about how others respond to you in social situations? (eliciting specific examples from experiments)*’; ‘*What have we learnt so far in treatment about acceptable you are as person?*’; ‘*Can you bring to mind an image of how you actually looked on the video?*’). Patients are then encouraged to reappraise the memory differently, e.g. ‘*If you knew another child aged nine (i.e. age patient was in the memory) who was being treated in this way, would you tell them it meant they were weird, weak and inferior? (add patient’s specific meanings). Why not? What would you say to them instead?*’. Patients are usually able to conclude that the actions of critical/bullying/prejudiced others did not mean anything negative about *them*, that they are an acceptable person in the world. This new updated information is then brought back into the memory through imagery rescripting carried out in three phases:

1. Through the eyes of the younger self in the memory experiencing the event in present tense;
2. Through the eyes of the adult self, observing the event happen to the younger self and intervening to bring in the updated information at key points. This might involve the older self showing the younger self some key discoveries they have made during their treatment, such as looking together at a still-image taken of the patient during a key behavioural experiment or reading other person feedback obtained in treatment;
3. Through the eyes of the younger self, who then experiences the older self-bringing the updated information in with compassion.

Imagery rescripting can be a powerful way to update negative self-images and impressions and achieve further reduction in persistent negative self-evaluations. In the weeks that follow present-day social situations are no longer being processed through the lens of the past and patients can accumulate more evidence to disconfirm their beliefs.

### Addressing maintaining strategies outside of social situations: worrying in advance and post-event rumination

All patients with SAD have negative self-evaluative thoughts about their social performance and some can spend hours worrying in advance of social situations or ruminating on perceived social mistakes after the event. These processes maintain negative self-evaluations as patients project their own thoughts into the minds of others. For example, a patient who spends many hours worrying in advance of a work lunch and then dwelling on her own perceived inadequacies afterwards, assumes that others are equally critical of her performance, and this fuels her belief that she is ‘boring and inadequate’. When ruminating about a particular event that has just happened patients often then link it to other times when they feel they have come across badly, creating a subjective log of multiple social failures and inadequacy. As a consequence, feeling that one came across as boring in a particular situation becomes an additional bit of evidence that one is a boring person.

For many patients worry in advance and post-event rumination significantly reduce as they discover, through behavioural experiments and video feedback, that they come across as acceptable and much better than they think, and that others are not as focused on them as they thought. The Social Phobia Weekly Summary Scale (SPWSS; available to download from the oxcadatresources.com website) includes items on post-event rumination and worry in advance that can help monitor these processes as treatment progresses. If therapists spot that either of these processes are not reducing, helping patients to notice, label and disengage from worry or rumination by reminding themselves of its many disadvantages, can be useful. A key message is that dwelling on social interactions or worrying in advance does not lead to reliable data about how the person comes across. The route to getting more reliable data is by turning their worry or self-critical thoughts into an experiment to put to the test. For example, a patient who thought he messed up when he presented at a work away-day decided rather than avoiding his colleagues afterwards and beating himself up, he would go and speak to them over the coffee break and see if they responded negatively towards him. To his surprise they were chatty and told him he did a good job.

### ‘You bloody idiot! You messed up again, you are useless’: additional manoeuvres for addressing self-criticism

Some patients with SAD experience their self-evaluative thoughts in a self-critical way, with a harsh and attacking tone. This is problematic as when these patients project their thoughts into the minds of others, it gives the impression that others are thinking of them in the same derogatory and attacking manner, leading to other negative emotions including shame and sadness. If this process is activated during therapy sessions it can become a roadblock to belief change if not addressed. Simple manoeuvres, interwoven with standard CT-SAD interventions, can often help patients get into a less critical mindset. For example, some patients become highly critical of their appearance when doing video feedback of an experiment (e.g. ‘I look awful, I look like a bloody idiot, my therapist must think I’m such a fool’). Therapists can help the patient recognise their thoughts as self-criticism, then switch off the video sound and/or covering the image of the patient so that they can only see the kind and friendly responses of the conversational partner.

Patients can believe that beating themselves up is a helpful way to keep themselves in check and avoid making social mistakes in future. In these cases, exploring the disadvantages of self-criticism can be helpful. For patients who find it difficult to identify any downsides to self-criticism, it can help to ask them to consider a child in their life and whether they would choose either a highly critical or a more compassionate teacher to guide this child’s learning. When considering somebody else, patients are usually able to see that a harsh, critical approach does not facilitate learning. These patients may benefit from enacting how their inner critic speaks to them after a perceived social mishap. Hearing aloud how the self-critic sounds and exploring how anybody might feel if they experienced this harsh self-attack can make it easier for patients to recognise its negative impact and spot their inner critic in future (Gilbert, 2010).

As patients tend to project their self-criticism into the minds of others, it is particularly important that when patients start to recognise their inner critic, they remind themselves ‘that’s my inner critic, not what others think!’ and then turn the content of the self-criticism into predictions to test in an experiment (the accurate data is in the moment, not in their head!). For those patients whose inner critic is an internalised person from their past (such as a critical parent), it can help to recognise that the critic is an echo from the past and label it as such (e.g. ‘*that is Dad’s voice, not what others think now*’). Patients whose critical voice adopts a particularly harsh tone may need to be encouraged to practise speaking to themselves with more kindness and give themselves credit for achievements, however small, as treatment progresses.

Occasionally people can find themselves in a highly critical environment at work or in the family. In cases such as this it is important to encourage them to explore social interactions outside of these contexts where they will have more of an opportunity to be appreciated. It can also be useful to use Christine Padesky’s assertive defence of the self (Padesky, 1997).

### Maximising change in persistent negative self-evaluations

Our experience is that if therapists ensure that all of the steps in CT-SAD include a focus on persistent negative self-evaluations when these are present, such evaluations steadily decrease as treatment progresses. Of course, one wants to maximise the extent to which the evaluations change over a full course of treatment. For this reason, we recommend specifically probing for any residual belief in such evaluations as one approaches the end of therapy. A range of interventions can then be brought together to deal with any lingering belief in the negative self-evaluations. We recommend re-administering the SBQ (Clark, 2005) to ensure that all safety behaviours have been addressed. Lingering use of safety behaviours can prevent complete change in negative self-evaluations. More general behavioural withdrawal may need targeting. For example, for some patients their negative self-evaluations and self-criticism hold them back from doing other things they enjoy (such as playing an instrument, drawing/painting, etc.). They may consequently live a restricted life which can in turn maintain their negative self-beliefs. It can be helpful to encourage patients to plan activities they may have previously avoided. For example, a patient who had always seen himself as inferior and unlikeable wrote a list of things he might do if he was 100% acceptable (e.g. invite a colleague to go for coffee, try a new restaurant I’ve not been to before, ask a friend to go on holiday with him, sign up for internet dating, start to play the guitar, start drawing again). He put these things in a jar and decided to pick one challenge a week. He found it helpful to keep a daily record of any evidence, however small, that he was acceptable on a traditional positive data log (see Padesky, 1994). These strategies were helpful to strengthen his view of himself as acceptable. The dichotomous nature of some beliefs could also be addressed using continua (see Padesky, 1994, for a description). Please note, in our experience cognitive techniques can have limited value when patients are continuing to use their safety behaviours as patients are more likely to discount evidence (e.g. ‘They only found me acceptable because I was putting on a front’).

### The therapy blueprint

Towards the end of CT-SAD we develop a therapy blueprint. The blueprint is a document summarising the patient’s key learning points from therapy and how they will continue to build on their learning in the booster phase and beyond. A template of a blueprint document can be found on the oxcadatresources.com website. The process of reviewing all the accumulated experiments and video feedback done throughout therapy can be a powerful way to pull together what patients have discovered about all their negative thoughts and beliefs. Patients’ discoveries about their persistent negative self-evaluations need to be incorporated into the blueprint.

### Co-morbid depression

Depression is a common co-morbidity in SAD (NICE, 2013) and one of the most frequent questions we are asked is when a patient presents with a diagnosis of both SAD and depression, what should be prioritised in treatment. The presence of some negative self-evaluative beliefs (such as ‘I am inadequate’ or ‘I am unlikeable’) does not help one make this decision because they can be present when either SAD or depression are the predominant problems.

#### Consider timelines

To help understand the inter-relationship between SAD and depression, it is helpful to establish the timeline for the onset of each condition. For example, ‘Did you have difficulty speaking to other people and feel socially anxious before you became depressed?’. If the answer is yes, then it is likely that the person has a primary social anxiety disorder that requires treatment. If the answer is that the person only started finding it difficult to speak to other people once they became depressed, then it is likely that the social concerns, including negative self-evaluations, are largely a reflection of the general loss of confidence that occurs in depression. In such situations it is often best to focus on initially treating the depression and then see if any social anxiety symptoms remain.

#### The magic wand question

In cases where the primary social anxiety is present and the person is also markedly depressed we find that it can be helpful to ask the magic wand question to determine whether the depression is secondary to the social anxiety or is an additional problem that requires treatment in its own right: ‘*If I had a magic wand, and if I waved it you would no longer feel anxious talking to other people, do you think you would still be depressed?*’. If the person says probably not or the depression would be very mild, then it is likely that the depression is secondary to SAD and that focusing directly on treating SAD is likely to lead to marked improvement in depressive symptoms as well. The only exception to this is if the secondary depression is so severe that the person would have difficulty engaging in any of the tasks in CT-SAD. If that is the case, it can be helpful to have a few sessions of behavioural activation or other depression-focused interventions to help start lift the patient’s mood and enable them to engage with the tasks involved in CT-SAD. If the person says that it would be great if the magic wand meant that they were no longer anxious talking to other people, but they still think that they would be severely depressed, then it is likely that the patient has both primary SAD and primary depression. Treatment will then need to focus on both. In the case of negative self-evaluations, it is likely that these will extend beyond an individual’s concerns about social interactions and will also include issues to do with other aspects of their life (for example, I am inadequate because I have made a mess of my marriage, or I have failed in my job and in providing for my family). A further consideration for treatment planning is whether there are any immediate risk issues that may need to be prioritised.

We hope that this paper has been helpful in clarifying the central role of persistent negative self-evaluations in SAD and describing how therapists can ensure these are ‘put on the table’ from the start of therapy and addressed using all standard experiential CT-SAD interventions.

## Data Availability

Data availability is not applicable to this article as no new data were created or analysed in this study.

## Key practice points

1. Persistent negative self-evaluations (such as ‘I’m unlikeable’, ‘I’m weird’, etc.) are a key cognitive feature of SAD.
2. They can be addressed using all the standard CT-SAD interventions, such as behavioural experiments and video feedback.
3. Unlike the cognitive behavioural treatment protocol for depression, these beliefs are addressed early in CT-SAD and throughout all therapy sessions.
